# Ecological and genetic basis of metapopulation persistence of the Glanville fritillary butterfly in fragmented landscapes

**DOI:** 10.1038/ncomms14504

**Published:** 2017-02-17

**Authors:** Ilkka Hanski, Torsti Schulz, Swee Chong Wong, Virpi Ahola, Annukka Ruokolainen, Sami P. Ojanen

**Affiliations:** 1 Metapopulation Research Centre, Department of Biosciences, University of Helsinki, PO Box 65, Helsinki FI-00014, Finland

## Abstract

Ecologists are challenged to construct models of the biological consequences of habitat loss and fragmentation. Here, we use a metapopulation model to predict the distribution of the Glanville fritillary butterfly during 22 years across a large heterogeneous landscape with 4,415 small dry meadows. The majority (74%) of the 125 networks into which the meadows were clustered are below the extinction threshold for long-term persistence. Among the 33 networks above the threshold, spatial configuration and habitat quality rather than the pooled habitat area predict metapopulation size and persistence, but additionally allelic variation in a SNP in the gene Phosphoglucose isomerase (*Pgi*) explains 30% of variation in metapopulation size. The *Pgi* genotypes are associated with dispersal rate and hence with colonizations and extinctions. Associations between *Pgi* genotypes, population turnover and metapopulation size reflect eco-evolutionary dynamics, which may be a common feature in species inhabiting patch networks with unstable local dynamics.

## Abstract (web summary)

Habitat fragmentation can lead to extinction even when some habitat remains. Here, the authors model the metapopulation dynamics of the Glanville fritillary butterfly and show that persistence depends on spatial configuration and quality of the habitat, as well as on genotype-associated dispersal rate.

Habitat loss and fragmentation are the main drivers of ongoing loss of biodiversity^1^,^2^,^3^, but ecologists have made only limited progress in predicting the consequences of habitat loss on population viability and extinction^4^,^5^ in quantitative terms, and in clarifying the additional effects of fragmentation^6^,^7^. It has been suggested that framing the question as a dichotomy between the effects of loss of pooled habitat area versus fragmentation *per se* may be misleading, and that we should instead start by considering the causal mechanisms that underpin species' responses to altered spatial structure of habitat^8^,^9^. One approach that does this but is applicable only to highly fragmented landscapes (defined below) is based on the measure of metapopulation capacity, which integrates the effects of habitat amount and spatial configuration (fragmentation) into a single number.

From the viewpoint of practical conservation, it would be critically important to be able to answer questions such as how much habitat is sufficient for long-term persistence of populations and species. The species-area relationship (SAR) (ref. 10) has been used to predict extinctions due to habitat loss^11^,^12^,^13^,^14^, but these predictions continue to be debated^15^. They only provide a rough baseline prediction at best, because SAR does not take into account the temporal population dynamics following habitat loss, and SARs completely ignore any fragmentation effects (but see refs 16, 17, 18). In brief, ecologists are challenged to produce more predictive models of the consequences of habitat loss and fragmentation. Given the great variation in the ecological circumstances in which species and communities occur, and the range of spatial scales from small experimental study plots^19^ to the continental^20^ and even global scale^21^, it might be advisable to develop analyses and models for more circumscribed situations rather than aiming for universal approaches.

One common situation in nature is represented by highly fragmented landscapes, in which the focal habitat accounts for only a small percentage of the total landscape area, of the order of 1%. Very large numbers of invertebrates, plants and fungi inhabit highly fragmented landscapes^22^, and human land use makes the environment of large numbers of yet other species increasingly fragmented^23^,^24^. In highly fragmented landscapes, the focal habitat occurs in small patches, or fragments, which are distributed in a more or less aggregated manner across the landscape. The classical metapopulation approach^25^, assuming frequent extinctions of small local populations and frequent establishment of new populations in the currently unoccupied patches, has been developed for species inhabiting highly fragmented landscapes.

The large metapopulation of the Glanville fritillary butterfly (*Melitaea cinxia*) in the Åland Islands in Finland is a model system for the study of the ecological, genetic and evolutionary consequences of habitat fragmentation^25^,^26^. The landscape consists of a very large network of dry meadows with the pooled habitat area covering about 1% of the landscape^27^. Here, we analyse data for 22 years, comprising 66,527 records of the presence or absence of the butterfly in 4,415 habitat patches distributed among 125 semi-independent networks. Our aim is to test the predictive power of a spatially realistic metapopulation model^28^ and to examine other factors apart from habitat area and fragmentation that may influence metapopulation size and persistence. In particular, we analyse the effects of habitat quality, and the effect of a well-studied candidate gene, Phosphoglucose isomerase (*Pgi*), on the butterfly metapopulation dynamics and metapopulation size.

We show that the majority of the habitat networks of the butterfly are below the extinction threshold. Metapopulation persistence and sizes of the networks above the extinction threshold can be predicted by spatial configuration, habitat quality and *Pgi* genotypes. In accordance with earlier work in other systems^29^,^30^, associations between *Pgi* genotypes, population turnover and metapopulation size show compelling evidence that demographic and genetic dynamics are closely coupled, leading to observable eco-evolutionary dynamics in real systems at landscape scales.

## Results

### Extinction threshold in fragmented landscapes

**Figure 1:**
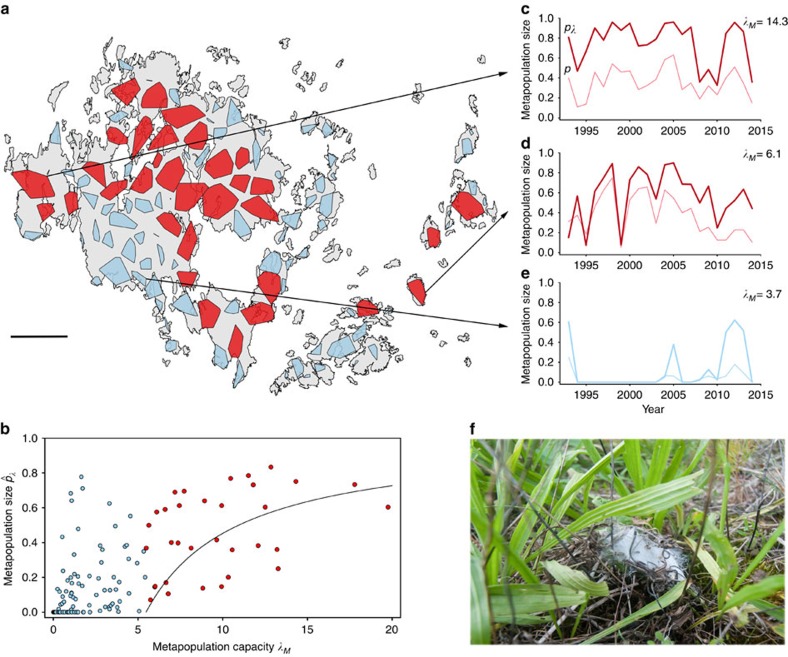
The study system and the metapopulation capacity of the habitat patch networks. (**a**) The red polygons demarcate habitat patches in the 33 networks above the extinction threshold (*δ*=5.47), while the 92 light-blue polygons are networks below the threshold (the smallest ones are not visible). The map contains shoreline data from the National Land Survey of Finland Topographic map 1:100,000 02/2015. Scale bar is 10 km. (**b**) Metapopulation size (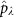) in each network as a function of metapopulation capacity (*λ*_*M*_). The continuous line gives metapopulation size as predicted by equation (1) parameterized with patch-level data using equation (2). Networks above and below the extinction threshold are shown by red and light-blue dots, respectively (*n*=125). (**c**–**e**) The three panels give time series of metapopulation size for three networks, one of which (**c**) is above the threshold, one is the island Sottunga (**d**) and the third one is a network below the threshold (**e**). The value of the metapopulation capacity is given for each network. The two lines are the fraction of occupied patches (thin line) and 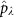 (thick line), which gives more weight to the dynamically more important patches. Sottunga was unoccupied in 1991, in which year the butterfly was translocated there. (**f**) A ‘winter nest' span by fifth instar larvae at the base of the host plant (*P. lanceolata*). The winter nests, inside which the larvae diapause, are sufficiently conspicuous to make the large-scale census of populations feasible. Photograph by Sami Ojanen.

The study system covers an area of 50 × 70 km (Fig. 1a), and consists of a large network of 4,415 dry meadows with one or both of the two host plant species of the butterfly^27^. The number of meadows that have been known to us and surveyed since the beginning of the study in 1993 has varied. Most importantly, the entire study area was re-mapped for the habitat in 1998–1999, which greatly increased the number of known meadows. For the analyses in which missing data would greatly affect the result, we have used the data set for the years 1999–2014.

Control surveys from the years 2009, 2011 and 2015 show that the presence of the butterfly is not detected in up to 15% of occupied meadows, but the non-detection only concerns meadows where the population is very small (Supplementary Tables 1–3). Almost half of the 29 non-detected populations consist of a single larval group while the mean size of the detected populations is eight. Thus the populations that are missed have a very small influence on the dynamics of the metapopulation as a whole.

There is much spatial variation in the density of meadows across the study landscape. The life-time movements of the butterfly are mostly limited to 2–3 km^31^. This means that, in any one generation, butterflies mostly move, mate and reproduce within areas that are less than 1% of the total landscape area. We have clustered the habitat patches into 125 semi-independent networks (Fig. 1a), which differ in terms of the number and spatial configuration of the patches and thus offer an opportunity to analyse the effects of landscape structure on the dynamics and distribution of species. Metapopulations inhabiting these networks are dynamically relatively independent from each other, though dispersal does occur between the networks and affects the dynamics of some of them, as will be demonstrated below. In the analyses, we use networks and the respective metapopulations as independent data points.

The variation in the number of patches in a network, and variation in the total amount of habitat (pooled area of patches) in a network explain only 9 and 11% of variation in the fraction of patches occupied, averaged across all years and denoted by *p* (Supplementary Figs 1 and 2). However, the fraction of patches occupied (*p*) is not a powerful measure of regional abundance, because it gives equal weight to small and large, and to well-connected and isolated habitat patches, which play very different roles in the dynamics of the metapopulation and hence have dissimilar consequences for long-term abundance^32^. Metapopulation theory^28^ suggests an alternative measure, *p*_*λ*_, which is a weighted average of patch occupancies, where the weights describe the role of individual patches in the dynamics of the metapopulation. In the deterministic model of patch occupancy dynamics^33^, the equilibrium value of *p*_*λ*_ is given by


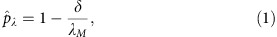


where *δ*=*e*/*c* is the ratio of the extinction and colonization rate parameters and is called the extinction threshold. We emphasize that the extinction threshold is a characteristic of the species, and hence the metapopulations in different networks are assumed to have the same value. *λ*_*M*_ is called the metapopulation capacity. This measure describes the features of the networks, and hence different networks in this study have different values. Mathematically, *λ*_*M*_ is the leading eigenvalue of matrix **M**, the elements of which describe patch-specific extinction and colonization rates. Metapopulation capacity integrates the effects of patch areas and their spatial locations on the capacity of the network to support a viable metapopulation^28^.

To apply equation (1) to data, we need to calculate the value of the metapopulation capacity (*λ*_*M*_) for each network, and to estimate the extinction threshold (*δ*) for the species. For the latter, there are two approaches, we may use an expression that gives the incidence of occupancy for each habitat patch at equilibrium, or we may use empirical data on observed extinctions and colonizations. The first approach is based on the equation giving the occupancy of patch *i* at equilibrium as 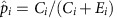, where *C*_*i*_ and *E*_*i*_ are the colonization and extinction rates. We make standard assumptions about how landscape structure affects *C*_*i*_ and *E*_*i*_, and add habitat quality in the model as explained in the Methods. We thereby derive the following equation for 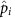



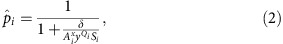


where 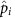 is the fraction of the 22 years that patch *i* has been occupied, the variables *A*_*i*_, *Q*_*i*_ and *S*_*i*_ are the area, quality and connectivity of patch *i,* respectively, and *x* and *y* are two parameters. The variable *Q*_*i*_ includes the abundance of host plants, the percentages of low and dry vegetation in the patch, and the percentage of the patch area that is grazed, all of which have highly significant effects on patch occupancy (Table 1). We fitted equation (2) to data from large patch networks with >50 patches and average patch occupancy >0.05, which are likely to have viable metapopulations. There are 24 such networks with altogether 2,330 habitat patches. The value of the extinction threshold thus estimated is *δ*=5.47, with the 95% confidence interval from 5.00 to 5.93.

The second approach is to estimate the values of the extinction (*e*) and colonization rate (*c*) parameters from data on annual extinction and colonization events, and calculating the extinction threshold as their ratio (*δ*=*e*/*c*). This approach gave the estimate *δ*=3.64 with the 95% credible interval (Cr.I.) from 3.15 to 4.22 (Table 2). Though the extinction threshold for the latter model is different, the viable networks predicted by the models agree with all but three cases with a posterior probability of more than 0.95. The metapopulation capacities are also highly correlated (0.97) (Supplementary Fig. 3). We use the former estimate below.

### Viable versus non-viable patch networks

We next use equation (1) to calculate 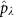 for the 125 networks into which the total of 4,415 habitat patches were clustered. In a linear regression, metapopulation capacity explains 40% of variation in the average of the annual 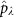 values (*F*-test: *F*_1,123_=84.16, *P*<10^−14^ ), which is substantially more than what the simple measures of network structure (patch number and pooled habitat area) explained of variation in the fraction of occupied patches (ca. 10%, above). More importantly, equation (1) predicts 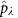 as a non-linear function of *λ*_*M*_ (Fig. 1b). The model fits the 33 networks above the extinction threshold (for an example see Fig. 1c) reasonably well (*r*=0.42, *t*-test: *t*_31_=2.68, *P*=0.015), though there is variation in 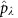, which to a large extent is due to a strong genetic effect (below). For the 92 networks with 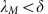, equation (1) predicts that the respective metapopulations are not viable, the predicted 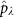 values are negative. Contrary to this prediction, however, many of these metapopulations are fairly large (Fig. 1b), though only temporarily so: 79 of the 92 networks (86%) were extinct (not a single occupied patch) for at least 5 years out of the 22 years (for an example see Fig. 1e). Presence of the butterfly in the ‘non-viable' networks may be due to dispersal from outside, which may rescue the metapopulation temporarily from permanent extinction. This hypothesis is supported by the present data: connectivity of the focal network to patches in the surrounding networks has a significant effect on metapopulation size (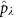) in the 92 non-viable networks (Supplementary Table 4).

Among the 33 networks above the extinction threshold, in a linear regression connectivity to the surrounding networks does not explain variation in 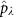 (*R*^2^=0.00, *F*-test: *F*_1,31_=0.07, *P*=0.80), and hence we conclude that the dynamics of these metapopulations are relatively independent, not much influenced by dispersal from outside. Only 5 of the 33 networks were extinct, or apparently extinct (due to non-detection), for at least 5 years out of the 22 years, which is a much smaller percentage (15%) than in the networks below the threshold (86%) (Pearson's *χ*^*2*^-test: *χ*^*2*^=55.1, *P*<0.0001). The island of Sottunga outside the main Åland (Fig. 1a) is an informative example. This island with an area of 9.2 km^2^ and 49 small patches with the pooled area of 9.1 ha was unoccupied in 1991, when 62 larval groups were translocated to the island from the main Åland Island. The introduced metapopulation has persisted ever since (Fig. 1d), though it has gone through bottlenecks, minimally with one occupied meadow in 1999. In Sottunga, *λ*_*M*_=6.1, which is slightly greater than the extinction threshold (5.47). The observed dynamics are hence consistent with the model prediction.

### Genetic effects on metapopulation size and persistence

**Figure 2:**
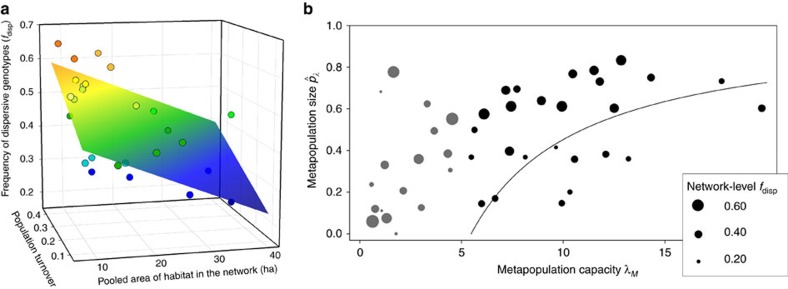
Association of dispersive genotypes with turnover and metapopulation size. (**a**) The frequency of the dispersive genotypes (*f*_*disp*_) in the SNP pgi:c.331A>C increases with the rate of population turnover but decreases with the pooled amount of habitat in the network. Turnover rate was calculated as the sum of the observed annual extinction and colonization events divided by the sum of the possible events. Multiple linear regression explains 38% of variation in *f*_*disp*_ (*F*-test: *F*_2,23_=8.42, *P*=0.0018, *n*=26). (**b**) The relationship between 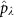 and metapopulation capacity (*λ*_*M*_) in the networks for which sufficient genetic data are available. The line shows the prediction from equation (1) with *δ*=5.47. The size of the symbol is proportional to *f*_*disp*_ as shown by the legend. Grey and black dots are for networks below and above the extinction threshold, respectively. Among the 26 networks above the threshold, multiple linear regression explains 45% of variation in metapopulation size (*F*-test: *F*_2,23_=10.82, *P*=0.0005). *P*=0.012 and 0.0006 (*t*-test: *t*_23_=2.74 and 3.92) for the regression coefficients of *λ*_*M*_ and *f*_*disp*_ , which alone in a simple regression explain 15% and 30% of the variation in metapopulation size, respectively (*F*-test: *F*_1,24_=3.913 and 11.13, *P*=0.06 and 0.003).

**Figure 3:**
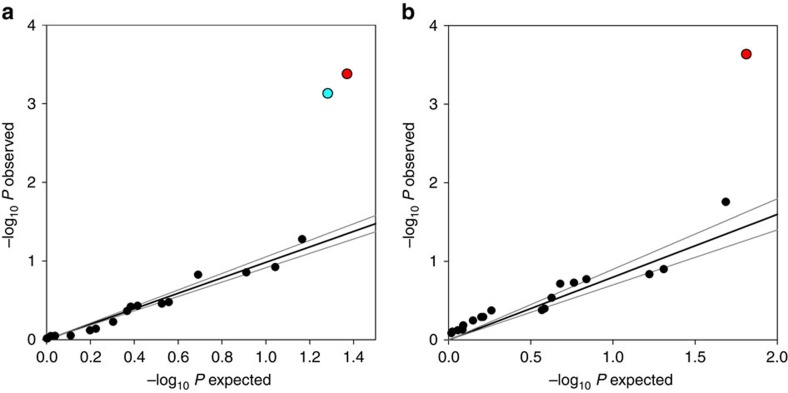
Association between allele frequency and population dynamics in patch networks. (**a**) The observed *P* value is for the *F*-test on the multiple regression explaining allelic frequency with the rate of population turnover and the pooled amount of habitat in the network, as in Fig. 2a for pgi:c.331A>C (*n*=19). The expected *P* values were drawn from a uniform distribution between 0 and 1. The two outliers are pgi:c.331A>C (red dot) and a SNP in the gene *G6pd* (cyan dot). The slope of the regression (excluding the outliers) is 0.98. The average and standard deviation of the regression slope in 100 replicate analyses were 0.95 and 0.25, respectively. (**b**) The observed *P* value is for the *t*-test of the effect of the SNP on metapopulation size as in Fig. 2b for pgi:c.331A>C. The outlier is pgi:c.331A>C (red dot) (*n*=19). The slope of the regression line (excluding the outlier) is 0.80. The average and standard deviation of the regression slope in 100 replicate analyses were 1.07 and 0.25, respectively.

The model above accounts for the effects of the spatial configuration and habitat quality on 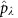. Previous work on the Glanville fritillary has shown that the single nucleotide polymorphism (SNP) pgi:c.331A>C in the glycolytic gene *Pgi* is significantly associated with flight metabolic rate (FMR) and dispersal rate in the field^31^,^34^. This is relevant here because dispersal and colonizations necessarily influence 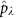. We characterize metapopulations in the 33 networks above the extinction threshold by the pooled frequency of the AC and CC individuals in the SNP pgi:c.331A>C, denoted by *f*_*disp*_. These butterflies have higher FMR and dispersal rate than the AA homozygotes. We use a large data set sampled across the entire study area in 2007–2012 and genotyped for pgi:c.331A>C as well as for 18 other SNPs (Supplementary Table 5). In the case of pgi:c.331A>C, this material includes a sample from >10 larval family groups per network for 26 of the 33 networks, with a median sample size of 68 individuals per network (Supplementary Data 1). Sample sizes are comparable for the other SNPs (Supplementary Table 5). In these 26 networks, *f*_*disp*_ increases with decreasing pooled area of habitat and with increasing rate of population turnover (Fig. 2a). The former is the product of the number of patches and the average patch area, and hence *f*_*disp*_ is high in networks with a small number of small patches (Supplementary Fig. 4). The effect of population turnover, the rate of extinctions and colonizations, is consistent with the idea that colonizations select for individuals with high dispersal capacity^35^. The results for the 18 other SNPs show that the significant associations involving pgi:c.331A>C are not due to population structure (Fig. 3a). The other significant SNP in Fig. 3a is from the gene Glucose-6-phosphate 1-dehydrogenase (*G6pd*), located next to *Pgi* in the glycolytic pathway, and which is significantly associated with the pooled area of habitat in the network (Supplementary Fig. 5), though not with population turnover. The major alleles in the two SNPs are negatively correlated at the network level (*r*=−0.48, *t*-test: *t*_17_=−2.26, *P*=0.037).

In the networks above the extinction threshold, metapopulation size 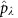 is significantly correlated with *f*_*disp*_ as well as with metapopulation capacity (Fig. 2b). In quantitative terms, 25% increase in *f*_*disp*_ from its median value, which corresponds to one standard deviation of the distribution of *f*_*disp*_ among the networks, increases metapopulation size by 17% (assuming the median value of *λ*_*M*_). This is a surprisingly large effect and not a result of inflation due to (cryptic) population stratification because none of the other SNPs show inflation (Fig. 3b). Moreover, *f*_*disp*_ is associated with metapopulation persistence: three viable networks (#4, #3 and #107) that were extinct or apparently extinct for at least 5 years had lower *f*_*disp*_ than the 27 networks that endured during the full 22 years (*t*-test: *t*_28_=2.33, *P*=0.028; includes those with a small sample size; genetic data were not available for 3 out of the 33 viable networks; Supplementary Data 1).

The results in Fig. 2a suggest that high population turnover in networks made up of a small number of small patches selects for highly dispersive butterflies (large *f*_*disp*_), which in turn increases metapopulation size (Fig. 2b). The latter effect follows from first principles, recalling that long-term metapopulation size is determined by a balance between extinctions and colonizations (equation (1), recall that *δ*=*e*/*c*). The probability of colonization of the currently unoccupied habitat patches increases, and the probability of extinction of the existing populations decreases, with *f*_*disp*_ in the surrounding populations from which immigrants arrive. We denote the latter variable by 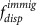 (Table 3). High 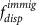 increases dispersal rate and thereby the colonization rate, and it increases the rescue effect and hence decreases the extinction rate in existing populations. Note that genetic change and demographic change are closely coupled: high *f*_*disp*_ in the network increases colonizations and decreases extinctions (Table 3), while extinctions and colonizations (population turnover) increase network-level *f*_*disp*_ (Fig. 2a).

## Discussion

Our results demonstrate how a large heterogeneous landscape is a mosaic of ‘hot' and ‘cold' sections in the sense that parts of the landscape harbour networks with viable metapopulations (viable networks for short), while the rest consists of non-viable networks in which the species is not expected to persist on the long term. In our case, 39% of the 4,415 habitat patches, and 29% of the pooled habitat area, are located in the cold sections, in networks below the extinction threshold. These networks may be temporarily occupied due to dispersal from the more favourable parts of the landscape, and these networks may thereby function as temporary stepping stones and facilitate the spread of a species across large areas. Classifying heterogeneous landscapes into hot and cold sections is helpful for practical conservation, as knowing the structure of the landscape helps direct conservation measures in a meaningful manner.

Our work shows how the landscape classification can be done in practice. To calculate metapopulation capacity, one needs to know habitat patch areas, connectivities and the average dispersal distance of the species. Additionally, further information is needed to estimate the scaling parameters influencing emigration, immigration and extinction rates. If such information is lacking, one may use values for other comparable species and knowledge about the biology of the focal species. One should note that while the value of the metapopulation capacity depends on these parameters, the ranking order of different networks is much less sensitive; and often it is useful to be able to rank networks from the most to the least viable. To give an idea of the empirical features of the 33 networks above the extinction threshold in the present study, they have minimally 32 habitat patches, minimally around 10 ha of habitat, and they minimally cover an area of 5 km^2^ (Supplementary Data 1). Though these figures do not apply as such to other systems, they can probably be used as a rough guide to the habitat requirements of other insect species of intermediate mobility that live in comparable habitat.

At the level of individual habitat patches, the probability of patch occupancy was best explained by patch areas and spatial locations, which are the ‘first order' effects in stochastic patch occupancy models. However, several features of habitat quality also made a substantial effect. This is to be expected, as there is always variation of quality in natural habitats across large areas. The result also depends on how the patches have been delineated in the first place. For instance, some potential patches may not be considered as patches at all because they are deemed to have such low quality. If they were nonetheless included among the habitat patches, the effect of habitat quality among the set of patches would be greater.

The amount of host plants and the percentages of dry and low vegetation had a positive effect on patch occupancy, while the percentage of grazed patch area had a negative effect. Desiccation of host plants, especially during the early larval instars in July, increases larval mortality and may even lead to local extinction. On the other hand, dry years are beneficial in the long term because they reduce the growth of grasses, which compete strongly with the host plants^36^. Grazing has similar conflicting short-term and long-term effects: grazing (trampling) has a direct negative effect on larval survival, but grazing also maintains habitat quality by preventing plant secondary succession^27^. There have been changes in summer temperature, precipitation and numbers of cattle, horses and sheep in the Åland Islands during the study period^27^, but these changes have not led to an increasing, nor decreasing, trend in the total metapopulation size of the Glanville fritillary^37^. On the other hand, the amplitude of fluctuations in the metapopulation as a whole has increased, reflecting increasing strength of regionally correlated environmental stochasticity in the dynamics^37^. These long-term changes have been attributed to increasing frequency of dry summers, a consequence of climate warming^37^.

The best predictive model at the network level has the metapopulation capacity as the explanatory variable describing the structure of the environment. This measure integrates the effects of the amount and spatial configuration of habitat, and hence there is no need (nor opportunity) to isolate the effects of the two. However, given that the total amount of habitat explains only 10% of variation in metapopulation size, while the metapopulation capacity explains 40%, it is clear that within the range considered the amount of habitat alone has little predictive power and that the spatial configuration (fragmentation) has an important effect. This is consistent with the general notion that fragmentation effects are increasingly important when the total amount of habitat in the landscape is small (here only about 1%)^7^,^38^.

An SNP in the gene *Pgi* has a surprisingly large effect in metapopulations inhabiting the viable networks, explaining 30% of variation in metapopulation size. This is a very large single-gene effect, but in qualitative terms the effect is expected based on previous results on *Pgi* in this species. The AC heterozygotes have higher FMR than the AA homozygotes by up to 50%, though difference between the genotypes is affected by interaction with body size and ambient temperature^31^,^34^,^39^. Butterflies with higher FMR have higher dispersal rate in the field^34^. More dispersive butterflies can be expected to be better colonizers, for which there are two types of evidence. First, results in Table 3 show directly that the colonization rate is increased by high frequency of *f*_*disp*_ among the butterflies in the surrounding source populations. Second, the dispersive butterflies are significantly more frequent in newly established than old local populations^35^. This comparison involves the F1 offspring of the actual colonizers, but we know that FMR has high heritability^40^. Finally, given that more dispersive butterflies increase the colonization rate and decrease the extinction rate (rescue effect) (Table 3), the balance between extinctions and colonizations is shifted towards higher metapopulation size (equation (1)). In summary, there are biologically consistent associations between the SNP pgi:c.331A>C and individual traits (flight metabolism, dispersal rate), population processes (colonizations, extinctions) and landscape-level metapopulation attributes (metapopulation size and persistence).

Apart from *Pgi*, the gene *G6pd* shows substantial polymorphism and significant association with landscapes structure (pooled area of habitat in the network). Genetic variation in metabolic enzymes, which often affect signalling pathways and have other moonlighting roles^41^,^42^, is surprisingly often associated with fitness-related traits^41^, but very little is known about the actual molecular functions. In *Drosophila,* polymorphism in *G6pd* and other enzymes around the glucose-6-phosphate (G6P) branching point of the glycolytic pathway often deviate from neutrality, suggesting that they may have been subject to adaptive evolution^43^,^44^.

Allelic variation in *Pgi* is associated with life-history traits in many insects and plants^45^, in which comparable results on population dynamics and size could be expected to occur. In other species living in highly fragmented landscapes, other heritable traits influencing dispersal and colonization may be selected for and be coupled with population dynamics in the same way as pgi:c.331A>C is in the Glanville fritillary. Examples range from gene expression profiles^46^,^47^ to wing polymorphism in insects^48^ and behavioural traits in vertebrates^49^. We suggest that species inhabiting patchy habitats often exhibit such eco-evolutionary dynamics^29^,^30^, reciprocal interaction between microevolutionary and demographic dynamics. A yet open question is how commonly eco-evolutionary dynamics increase the persistence of populations^50^. Our results provide a convincing example, as *f*_*disp*_ strongly affects metapopulation size and as metapopulations close to the extinction threshold are more persistent if they have high *f*_*disp*_. Selection thus compensates, to a limited extent, for the adverse consequences of habitat loss.

## Methods

### Habitat patches and patch networks

In the Åland islands, the Glanville fritillary inhabits dry meadows that have at least one of the two larval host plant species, the ribwort plantain (*Plantago lanceolata*) or the spiked speedwell (*Veronica spicata*)^25^,^36^. The habitat patches are small: the median size is 0.06 ha, and only 1% are greater than 2 ha. We have mapped the entire Åland Islands, an area of 50 × 70 km, for the habitat patches during two periods, in 1993 and 1998–1999. The first survey yielded ca. 1,500 patches, while the total number after the second survey has been ca. 4,500 patches, including a large number of very small ones. The exact current number is 4,415 (September 2015). We census the caterpillars annually in the overwintering stage by visiting all the habitat patches in late summer with the help of 50–70 field assistans^27^. Hence, the census results reflect the presence of local breeding populations. The census is possible because caterpillars overwinter in groups of ca. 100 of mostly full-sibs under a relatively conspicuous silken web woven at the base of the host plant (Fig. 1f). The locations of the found groups are marked with GPS and data is stored into the database in the field. A sample of three living larvae is collected from each group (1995, 2002, 2007–2012) or a subset of groups (2013->) and taken to a butterfly rearing facility for further studies. Because only a third of the patches were censused before 1998–1999, the number of presence/absence records for individual years and populations is less than 22 × 4,415. Additionally, there is a small amount of missing data especially from isolated low-quality networks that have remained completely unoccupied during the entire study period.

The 4,415 patches were divided into 125 sub-networks using the software SPOMSIM (ref. 51). The construction of patch networks is based on geometric average linkage clustering using connectivity (see ‘Connectivity of habitat patches' below) as the distance measure and selecting a level of clustering that produced networks within which individual patches can be easily reached by dispersing butterflies, while movements between networks would be uncommon^52^. In the clustering, we used the same parameter values as Moilanen^51^: *α*=1.0, *b*=0.5 and *q*=1.5, where *α* is the parameter of the negative exponential dispersal kernel (see ‘Connectivity of habitat patches' below) and the other parameters control the clustering procedure. Connectivity of the networks to each other is lower in reality than this calculation suggests, because the clusters of patches (networks) are often separated dispersal barriers, such as tracts of forest. These landscape effects are not taken into account in the clustering algorithm, based on physical distances only.

### Analysis of control survey data

Previously the probability of not recording an existing population (non-detection) has been estimated to range from 0.1 (ref. 36) to 0.28 (ref. 27). In this study, we estimate the prevalence of not detecting a larval group and not detecting a patch as occupied (Supplementary Tables 1–2) using 311 control visits from the years 2009, 2011 and 2015. We also study the relationship of non-detection to local population size (Supplementary Table 3).The control data were only collected in networks where at least some larval groups were found during the main surveys. Out of 200 occupied patches, the control visits found 29 populations that were not recorded during the main survey. We assume that the remaining 111 patches were truly unoccupied, and therefore they are excluded from the following analyses.

The statistical analysis of control survey data consists of three generalized linear models. In the first model (Supplementary Table 1) we estimate the probability of detecting a larval group with a binomial regression model with a logit-link function. As the dependent variable we use the proportion of larval groups found during regular survey, and as explanatory variables log-transformed patch area (ha) and total number of larval groups. The first explanatory variable tests for the dependence of detecting a larval group on patch area, and the second for the dependence of detecting a larval group on total number of larval groups. As larval groups are not randomly distributed within patches, we expect larval groups to be more easily found when they occur in larger numbers.

In the second model (Supplementary Table 2) we analyse the overall probability of not detecting a patch as occupied when it is occupied. The dependent variable of the logistic regression is the binary indicator of non-detection, with an occupied patch being detected during the main survey coded as 0 and not detected as 1. The model uses the logit-link function. The explanatory variables are log-transformed patch area (ha) and total number of larval groups.

In the third model (Supplementary Table 3) we analyse the difference in population sizes between detected and non-detected populations using Poisson regression analysis. The dependent variable is the number of larval groups. The explanatory variables are log-transformed patch area (ha) and the binary variable for non-detection explained above.

All models were estimated with the Bayesian modelling package *rstanarm* in R (ref. 53). For intercepts and predictors we use Student's *t* distribution with mean zero and four degrees of freedom as the prior distribution. The scale of the prior distribution is 10 for the intercept and 2.5 for the predictors. Each model was run with four chains for 1,000 warm-up and 1,000 sampling steps. For all parameters in all three models, the number of effective samples was >1,000, the convergence measure 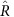 was <1.005, and the Monte Carlo standard error of the parameter means was <0.015.

### Habitat patch quality

Habitat patch quality is assessed during the census of larvae in late summer^27^. Abundance of both host plants is estimated separately on the scale from 0 to 3. As both of the host plant species perform better when growing in low vegetation and where competitively superior grasses are less common, we estimate the percentage of host plant areas surrounded by low vegetation. We also estimate the percentage of desiccated host plants in the population and finally, we estimate the percentage of patch area under grazing.

### Connectivity of habitat patches

Connectivity of patch *i* is a proxy for the number of immigrants arriving at patch *i* during one generation (year). Connectivity *S*_*i*_ is calculated as 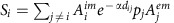, where *A*_*j*_ and *A*_*i*_ are the areas of the source (*j*) and target (*i*) patches (in ha), *p*_*j*_ is the incidence of occupancy of source patch *j*, *d*_*ij*_ is the distance between patches *i* and *j* (in km), and 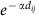 is the negative exponential dispersal kernel with parameter *α* (refs 33, 54). The incidence of occupancy *p*_*j*_ has the value of 1 for occupied and 0 for unoccupied patches, or a value between 0 and 1 if the average value of *p*_*j*_ across several years is used. The exponents *im* and *em* scale the rates of immigration and emigration by patch area. We estimated the values of *im*, *em* and *α* using the data from the years 1999 to 2014 (Table 2). The estimated values for *im* and *em* (*im*=0.44, *em*=*0.22*) are within the range of previous results for the Glanville fritillary and the False heath fritillary, a closely related butterfly with similar ecology^55^. The estimated value for *α*=0.93 agrees with earlier studies based on mark-recapture data^56^. In the model based on equation (2) and linear models where *α* and *em* cannot be estimated independently (see ‘Modelling extinction and colonization events' below), we assume *α*=1 and *em*=0.2 based on the results above.

### Colonization and extinction rates

Colonization rate of patch *i* is given by *cS*_*i*_, where *c* is the colonization rate parameter. Extinction rate is assumed to depend on the area *A*_*i*_ and quality *Q*_*i*_ of patch *i* as 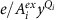, where *e, ex* and *y* are parameters estimated from the present data (Table 2). The construction of the habitat quality variable *Q*_*i*_ is described in the section ‘Parameter estimation based on equation (2)' below.

### Connectivity of patch networks

The nearest patch in other networks is located 1.75 km on average from the centre point of the focal network (minimum and maximum 0.62 and 5.13 km, respectively, *n*=125), while the average distance to the ten nearest other patches is 2.17 km (0.95 and 6.12 km, respectively). Though most butterflies move <1 km in their life-time, a substantial fraction, on the order of 10%, fly a distance of 2 km or more^31^,^56^,^57^, and hence we can expect some movements between the networks. We calculated a measure of connectivity for each network as 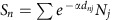, where *N*_*j*_ is the average population size (number of larval groups) in patch *j* across the years, and *d*_*nj*_ is the distance in km between patch *j* and the centre point of network *n*. The sum is taken over all patches *j* that do not belong to network *n*. We assume *α*=1 as in the calculation of connectivity for individual habitat patches.

### Metapopulation capacity

Based on the assumptions of how patch areas, qualities and spatial locations in the network influence the extinction and colonization rates, one may construct an *n* by *n* matrix **M** for a network with *n* patches^33^,^58^. The leading eigenvalue of **M** is called the metapopulation capacity and denoted by *λ*_*M*_. In the present case, the elements of **M** are *m*_*ii*_=0 and 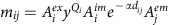, where 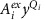
is proportional to the expected life-time of population *i* (the inverse of extinction rate; see ‘Colonization and extinction rates' above), while the remaining terms in *m*_*ij*_ come from the assumptions of how connectivity depends on patch areas and configuration (see ‘Connectivity of habitat patches' above). Metapopulation capacity integrates the effects of patch areas, qualities and their spatial locations on the capacity of the network to support a viable metapopulation^28^,^33^.

### Parameter estimation based on equation (2)

We estimated model parameters with non-linear regression using the expression for the probability of patch occupancy at equilibrium^33^,^54^. Parameter estimation was carried out in two stages. In the first stage, we used equation


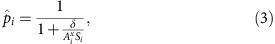


where *δ*=*e*/*c* is the ratio of the extinction and colonization rate parameters, called the extinction threshold, and *x* is the sum of the exponents *ex* and *im* (see definition of *m*_*ij*_ in the section ‘Metapopulation capacity' above). 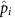 is the observed frequency of occupancy of patch *i* during the 22 years. Equation (3) was fitted to data from large networks with >50 patches and average 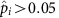, which are likely to have viable metapopulations. There are 24 such networks with 2,330 habitat patches. The estimated parameter values are *δ*=3.91 and *x*=0.51.

We next used equation (3) to calculate the predicted values of 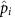 for each patch in the 24 large networks. Using logistic regression, we then explained the observed values of 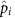 with the predicted values as well as with four attributes of patch quality. Given that the effects of the four quality variables are of the same order of magnitude (Table 1), we summarized their effects with the term 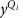, where *y* is a parameter and 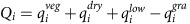, the sum of the four quality variables for patch *i,* all rescaled to the interval [−1..1] (the sign of 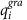
is reversed because large values correspond to low quality; Table 1). Adding habitat quality with this term into the model, equation (3) is turned to equation (2) in the Results, which is repeated here


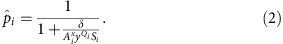


In the second step, we fitted equation (2) to the data from the 24 large networks, and obtained the parameter values *δ*=5.47 (95% confidence interval 5.00–5.93), *x*=0.428 (0.395–0.462) and *y*=1.71 (1.63–1.79). Equation (2) with these parameter values predicts the observed patch occupancy as well as the logistic model with all the four patch quality variables added separately (Table 1). Therefore, we conclude that adding the term 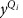
into the model is a simple and effective way of taking several features of habitat quality into account in the context of the present model.

### Parameter estimation from colonization and extinction events

We also estimated model parameters using data on annual extinction and colonization events in all networks using the data for the years 1999–2014 (Table 2). The discrete-time extinction and colonization probabilities are given by 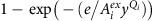 and 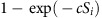, respectively (see ‘Colonization and extinction rates' and ‘Connectivity of habitat patches' above). The model was implemented using Stan version 2.8, and the parameters were estimated using Hamiltonian Markov Chain Monte Carlo^59^. All parameters had uniformly distributed priors on the positive real numbers. The model was run with four chains for 1,000 warm-up steps and 1,000 steps of sampling. For all parameters the number of effective samples was >1,300, the convergence measure 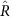 was <1.004 and the Monte Carlo standard error of the parameter means was <0.002.

### Modelling extinction and colonization events

Colonizations and extinctions were analysed using a varying intercepts hierarchical logistic regression model with habitat patch and annual network-level random effects (Table 3). We used data for the years 1999–2014 in the analysis, because there is much missing data for the earlier years (see ‘Habitat patches and patch networks' above). We also repeated the analysis for the years 1993–2014 and 2007–2014 with very similar results and the same conclusions (genetic data were collected in 2007–2012; see ‘Genetic data and measures' below).

The patch-specific colonization and extinction events were estimated using patch area *A*^0.2^, connectivity *S*_*i*_ and 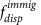
as predictors. The structure of the regression model is given by


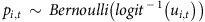



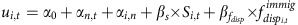



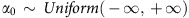



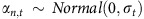



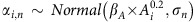



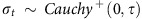



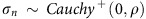



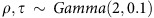



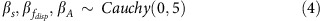


where *p*_*i,t*_ indicates the colonization or extinction event in patch *i* in year *t*, *α*_0_ is the mean extinction or colonization rate, *α*_*n,t*_ is the random intercept for network *n* in year *t*, and *α*_*i,n*_ is the random intercept of patch *i* in network *n*. The annual network effects account for network structure and the variation in the scale of spatial synchrony between years. The network-specific patch effect accounts for differences between networks and repeated sampling of patches. *β*_*S*_, *β*_*fdisp*_ and *β*_*A*_ are the coefficients for annual connectivity, annual frequency of dispersive immigrants and patch area. Patch area (in ha) is scaled to power 0.2. The exponent 0.2 scales expected population size and the number of immigrants and emigrants by patch area, which approximates the parameters *ex*, *im* and *em* (see ‘Connectivity of habitat patches' above). The predictors *A*^0.2^, *S* and 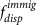 are centred to zero mean and unit standard deviation to assist the comparison of their relative effects and to ensure computational stability. The models were implemented in Stan 2.8 and estimated using Hamiltonian Markov Chain Monte Carlo and the No-U-turn sampler for 1,500 warm-up and 1,500 sampling steps with four Markov chains^59^.

### Genetic data and measures

During the years 2007–2012, Glanville fritillary larvae were sampled from natural populations across the Åland Islands in the context of several studies^60^,^61^,^62^,^63^. These studies have produced a large database of SNP data that we use here. Out of ca. 300 SNPs, there are 19 SNPs with large samples from most of the viable networks, in which *λ*_*M*_>*δ* (Supplementary Table 5). These SNPs, including pgi:c.331A>C, which is the candidate gene in the present study, have been genotyped with the Sequenom (SEQUENOM Inc., San Diego, CA, USA) and KASP (LGC, Teddington, UK) platforms, and the data are managed with the Progeny database (Progeny Software, Delray Beach, FL, USA). DNA samples for both Sequenom and KASP genotyping were extracted with Nucleo Spin 96 Tissue kit (MACHEREY-NAGEL GmbH & Co. KG, Düren, Germany) in the Institute of Biotechnology, University of Helsinki. For technical details of genotyping see refs 60, 64 for Sequenom and ref. 62 for KASP. In the case of pgi:c.331A>C, we genotyped 979 individuals using both Sequenom and KASP, of which 968 had the same genotype call with both platforms (cross-platform concordance 98.9%). Most samples consist of triplets of larvae sampled from the same larval groups. To have an adequate sample for each of the 19 SNPs from each network included in the analysis, we required that individuals from more than 10 larval groups had been genotyped for a particular SNP from a particular network. For sample sizes see Supplementary Table 5 and Supplementary Data 1. The 18 SNPs apart from pgi:c.331A>C were used to control for population structure influencing associations involving pgi:c.331A>C.

In pgi:c.331A>C, the AC heterozygotes have higher dispersal rate than the AA homozygotes^34^,^65^,^66^. The CC homozygotes are less common than expected from the HW equilibrium in the Åland Islands, possibly because there is a common haplotype in which the C allele is linked with a recessive lethal mutation^66^. In this case, the uncommon CC homozygotes (7% in the present material) represent individuals that possess another haplotype. Previous empirical work has mostly compared the common AC and AA genotypes^34^,^67^,^68^,^69^. The few CC homozygotes for which FMR has been measured show comparable results than the AC individuals and higher FMR than the AA homozygotes^70^,^71^. Therefore, we assume that the AC and CC butterflies have comparable dispersal rate in the field, and we characterize metapopulations in the different patch networks by the pooled frequency of the AC and CC genotypes, denoted by *f*_*disp*_. We repeated the analyses assuming additive allelic effects and thus using the frequency of the C allele instead of *f*_*disp*_. The results were qualitatively similar and all the conclusions were the same as those from the dominance model. For the other SNPs, for which detailed results on FMR and different genotypes are not available, we assumed the additive model.

In the analysis in Table 3, we calculated the pooled frequency of the dispersive AC and CC genotypes in the source populations from which the migrants originated as 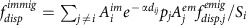, where *S*_*i*_ is the connectivity of patch *i* (see ‘Connectivity of habitat patches' above). 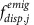 is the pooled frequency of the dispersing genotypes among the emigrants from patch *j*, calculated as 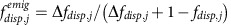, where *f*_*disp*, *j*_ is the corresponding genotype frequency among the residents in patch *j* (ref. 35). Following ref. 35, we assume Δ=2, and hence the formula assumes that the dispersive genotypes emigrate twice as fast as the AA homozygotes from the source populations. Note that we have a single estimate of 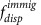, based on the data sampled in 2007–2012, and hence we have to assume that the genotype frequencies have remained relatively stable at the network level for the period 1993–2014, making the results in Table 3 conservative.

### Linear regression analyses

Linear regression models in Figs 2 and 3, Supplementary Figs 1–5, Supplementary Table 4, and those described in the Results section ‘Viable versus non viable networks' were carried out in R 3.3 (ref. 72). The significance of models was tested with an F-test and significance of predictors with a two-sided Student's *t*-test. Heteroscedasticity of residuals was studied with a studentized Breusch-Pagan test using the R package *lmtest* (ref. 73). The sensitivity of linear regression analyses to other deviations from model assumptions, such as outliers, was tested by comparing coefficient estimates of the linear regression model to those from a robust linear regression model estimated with a Huber M-estimator using the R package *MASS* (ref. 74). None of the fitted models showed violations to heteroscedasticity or other model assumptions.

### Data availability

Genetic variant data that support the findings of this study have been deposited in dbSNP with the accession codes ss2137343739 - ss2137343816. All other data that support the findings of this study are available from the corresponding author on reasonable request.

## Additional information

**How to cite this article:** Hanski, I. *et al*. Ecological and genetic basis of population persistence of the Glanville fritillary butterfly in fragmented landscapes. *Nat. Commun.* **8,** 14504 doi: 10.1038/ncomms14504 (2017).

**Publisher's note:** Springer Nature remains neutral with regard to jurisdictional claims in published maps and institutional affiliations.

## Supplementary Material

Supplementary Information Supplementary Figures and Supplementary Tables

Supplementary Data 1 Data for the 125 patch networks.

## Figures and Tables

**Table 1:** Logistic regression model for the average incidence of occupancy in the habitat patches in 24 large networks across 22 years.

Variable	estimate	s.e.	z	*P* value
Intercept	−3.70	0.06	−61.3	<10^−15^
Predicted *p*_*i*_	5.07	0.09	57.2	<10^−15^
Amount of host plants	1.00	0.05	19.4	<10^−15^
Percentage dry	3.15	0.22	13.8	<10^−15^
Percentage grazed	−1.00	0.06	−17.6	<10^−15^
Percentage low	0.50	0.10	4.7	<10^−5^

The explanatory variables are the predicted patch occupancy, calculated with equation (3) and *δ*=3.91 and *x*=0.51. The patch quality variables are described in the Methods section ‘Habitat patch quality'. Parameters were estimated with maximum likelihood using the logit-link function. Adjusted *R*^*2*^=0.56 for the full model, while *R*^*2*^=0.45 for the model with the predicted *p*_*i*_ as the only explanatory variable. Finally, *R*^*2*^=0.55 for the model in which the predicted *p*_*i*_ was calculated with equation (2), including the effect of habitat quality via the term 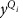
(Methods section ‘Parameter estimation based on equation (2)'). Note that the latter model explains the observed incidences of patch occupancy nearly as well as the model in this table.

**Table 2:** Estimated parameter values for the annual probabilities of extinction and colonization.

Variable	mean	s.d.	95% Cr.I
*a*	0.93	0.03	0.88–0.98
*y*	1.40	0.03	1.35–1.42
*ex*	0.23	0.01	0.21–0.26
*im*	0.44	0.01	0.42–0.47
*em*	0.22	0.03	0.15–0.28
*e*	0.38	0.01	0.36–0.41
*c*	0.11	0.01	0.09–0.12

Note that *δ*=*e*/*c*=3.64 (95% Cr.I. from 3.15 to 4.22). The numbers of networks above the extinction threshold is 31 at a posterior probability of at least 0.95.

**Table 3:** Logistic regression model for local extinctions and colonizations.

Variable	Colonizations			Extinctions		
	median	95% Cr.I.	odds ratio	median	95% Cr.I.	odds ratio
*α*_*0*_	−3.43	−3.56 to −3.30		−0.03	−0.21–0.15	
*τ*	1.50	0.87–2.62		1.38	−0.79–2.42	
*ρ*	0.68	0.50–0.92		0.66	0.44–0.95	
Patch area	0.76	0.70–0.82	2.14	−0.78	−0.86 to −0.70	0.46
Connectivity	0.81	0.73–0.89	2.25	−0.50	−0.62 to −0.38	0.61
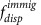	0.24	0.15–0.34	1.28	−0.13	−0.23 to −0.10	0.87

The numbers of extinction and colonization events out of possible events for the years 1999–2014 are 3,641/9,096 and 3,581/40,438, respectively. The explanatory variables are scaled patch area *A*^0.2^, connectivity *S* and the pooled frequency of the dispersive genotypes among the immigrants 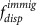. The intercept *α*_0_ is an estimate of the mean extinction or colonization rate (on the logit scale), *τ* is the estimated scale of annual network effects and *ρ* is the estimated scale of network-specific patch effects. Thus *τ* and *ρ* determine the magnitude and variance of the patch and network level random effects, respectively.
