# Supplementary material for: Ecological and genetic basis of metapopulation persistence of the Glanville fritillary butterfly in fragmented landscapes
Source: Nat Commun. 2017 Feb 17;8:14504. doi: 10.1038/ncomms14504 (PMC5321745; doi:10.1038/ncomms14504)
Supplement: Supplementary Information — Supplementary Figures and Supplementary Tables [file ncomms14504-s1.pdf]

Supplementary Figures

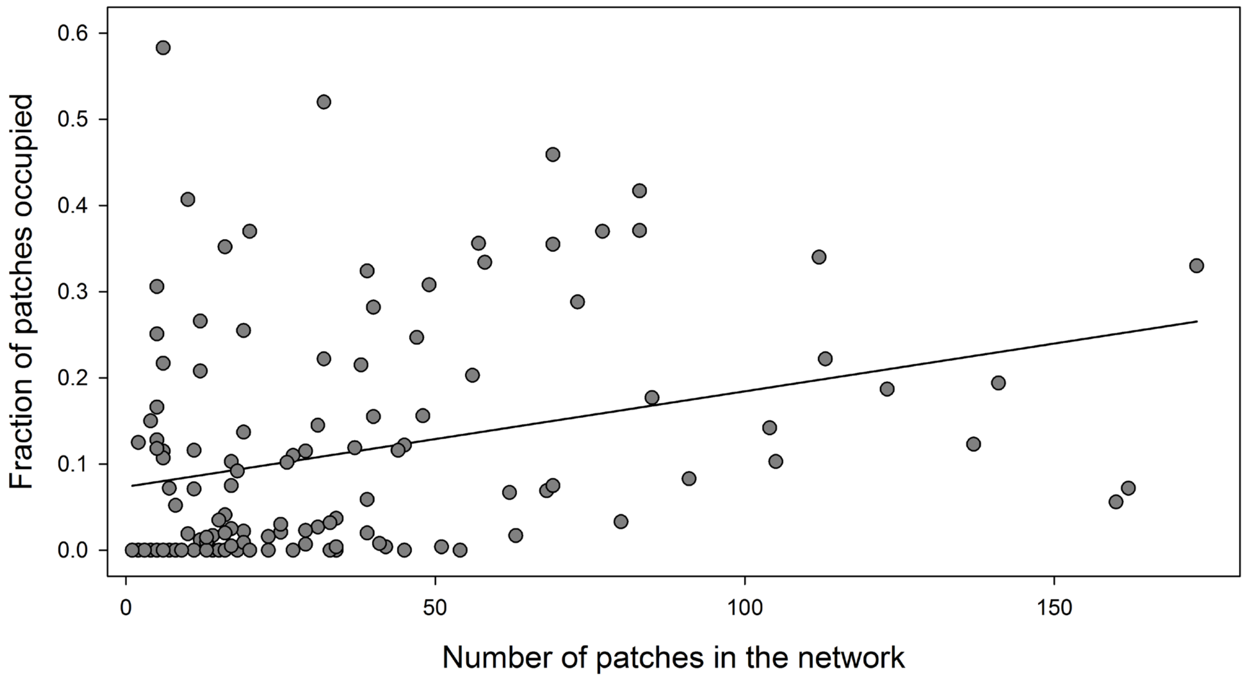

Supplementary Figure 1. Linear regression of the fraction of occupied habitat patches (*p*) against the number of patches in the network. The regression explains 9% of variation in *p*. The regression coefficient has $P = 0.0005$ (t-test: $t_{123} = 3.526$, $n = 125$).

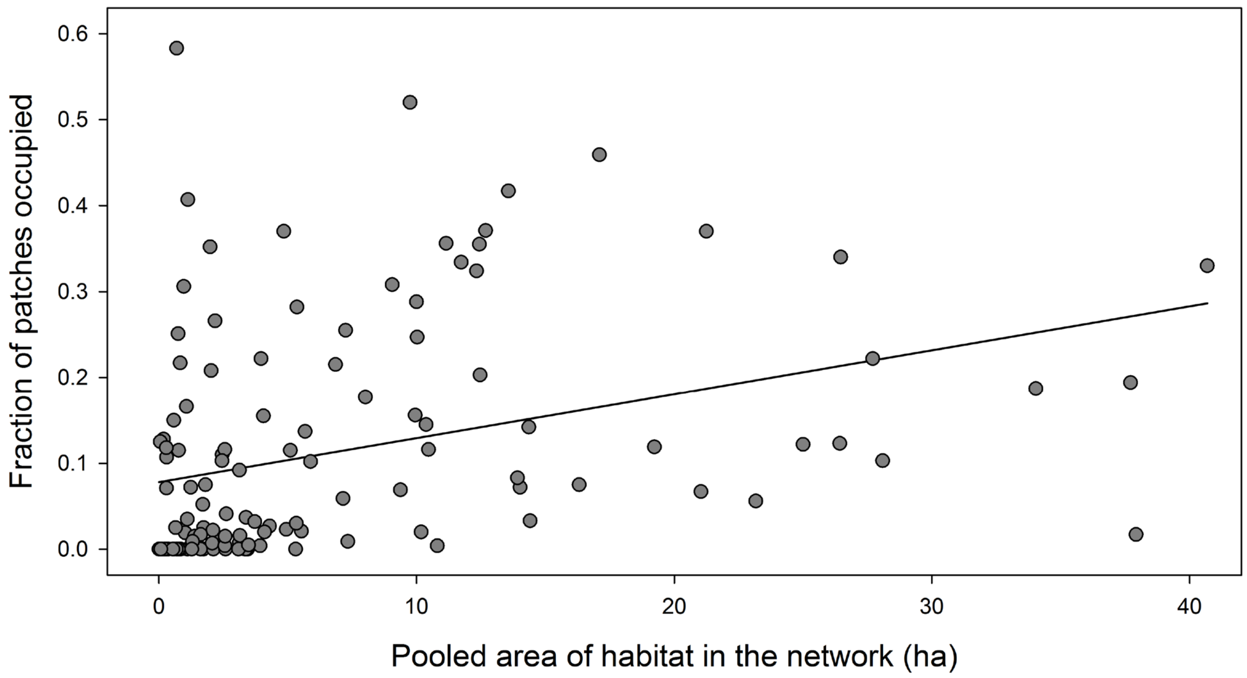

Supplementary Figure 2. Linear regression of the fraction of occupied habitat patches (*p*) against the pooled area of habitat in the network. The regression explains 11% of variation in *p*. The regression coefficient has $P = 0.0001$ (t-test: $t_{123} = 3.943$, $n = 125$).

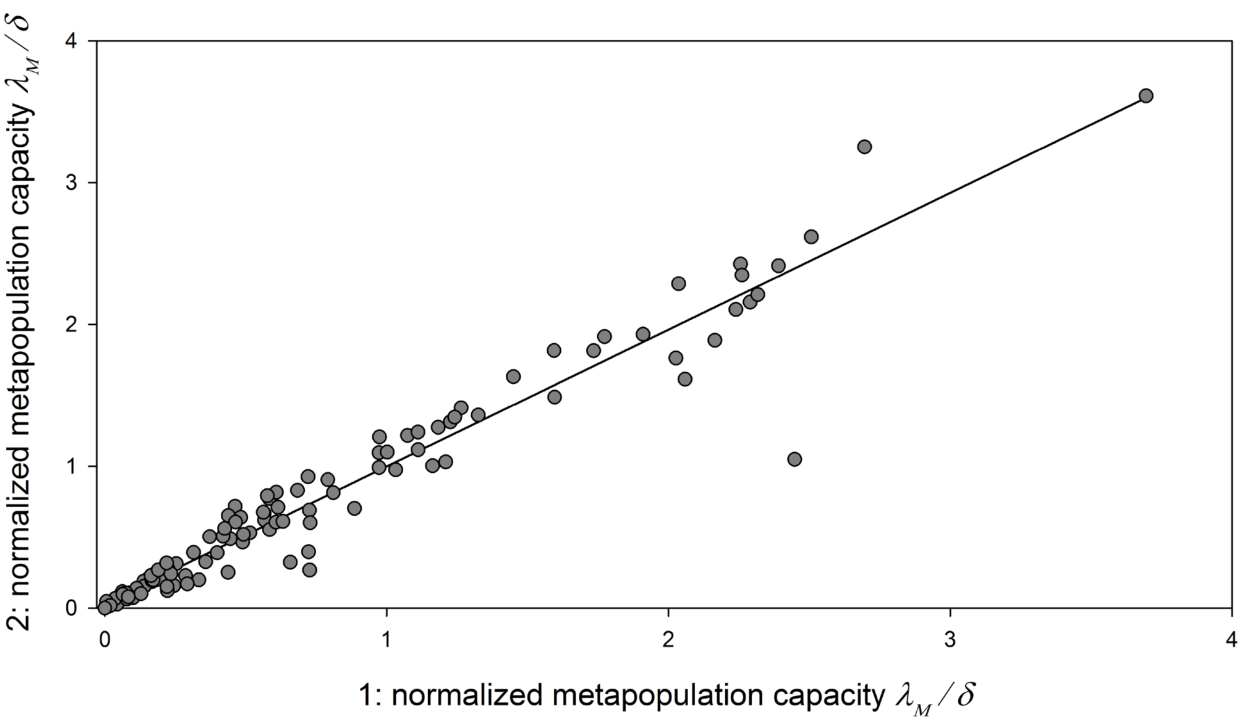

Supplementary Figure 3. Comparison of normalized metapopulation capacities from models based on 1: annual colonization and extinction events and 2: equation (2). The metapopulation capacity λ_M estimates are normalized by dividing them by the extinction threshold parameter δ estimated in the corresponding model. In a linear regression the metapopulation capacity of model 1 explains 94 % of variation in the metapopulation capacity of model 2 (F-test: $F_{1,123} = 1969$, $P < 10^{-15}$, $n = 125$). When the metapopulation capacities are normalized, neither the intercept nor the slope of the linear regression are significantly different from zero (t-test: $t_{123} = 1.50$, $P = 0.14$, $n = 125$) and one (t-test: $t_{123} = -1.62$, $P = 0.11$, $n = 125$) respectively.

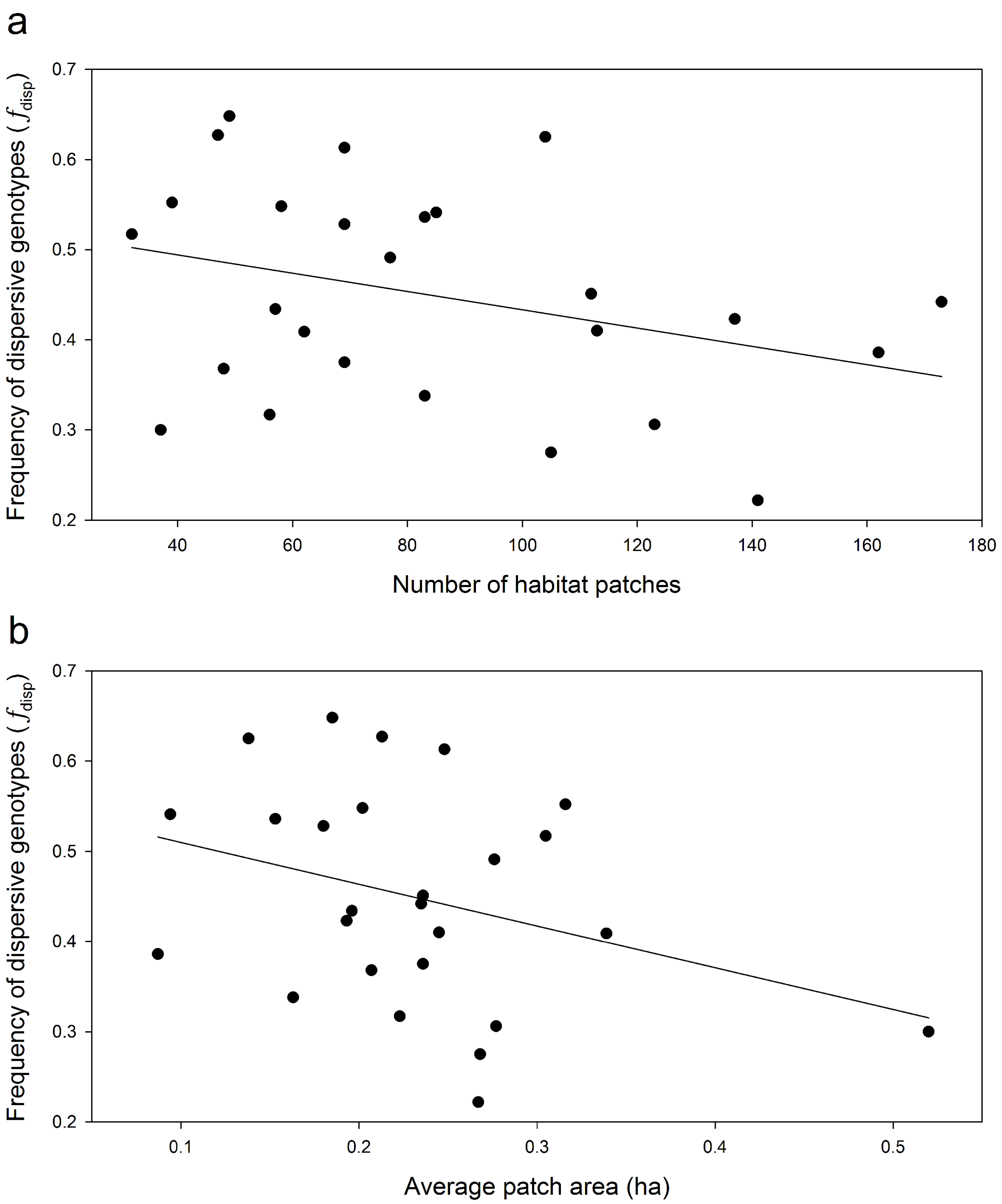

Supplementary Figure 4. Effects of the number of patches and the average patch area in the network on f_{disp}, the frequency of the dispersive genotypes in pgi:c.331A>C. a, f_{disp} decreases with increasing number of habitat patches in the network. **b,** f_{disp} decreases with increasing average size of the patches in the network. These two effects are not significant on their own, but they are both significant (*F*-test: $F_{2,23}$= 5.969, $P = 0.01$, $n = 26$) in a multiple regression model, which explains 28% of variation in f_{disp}.

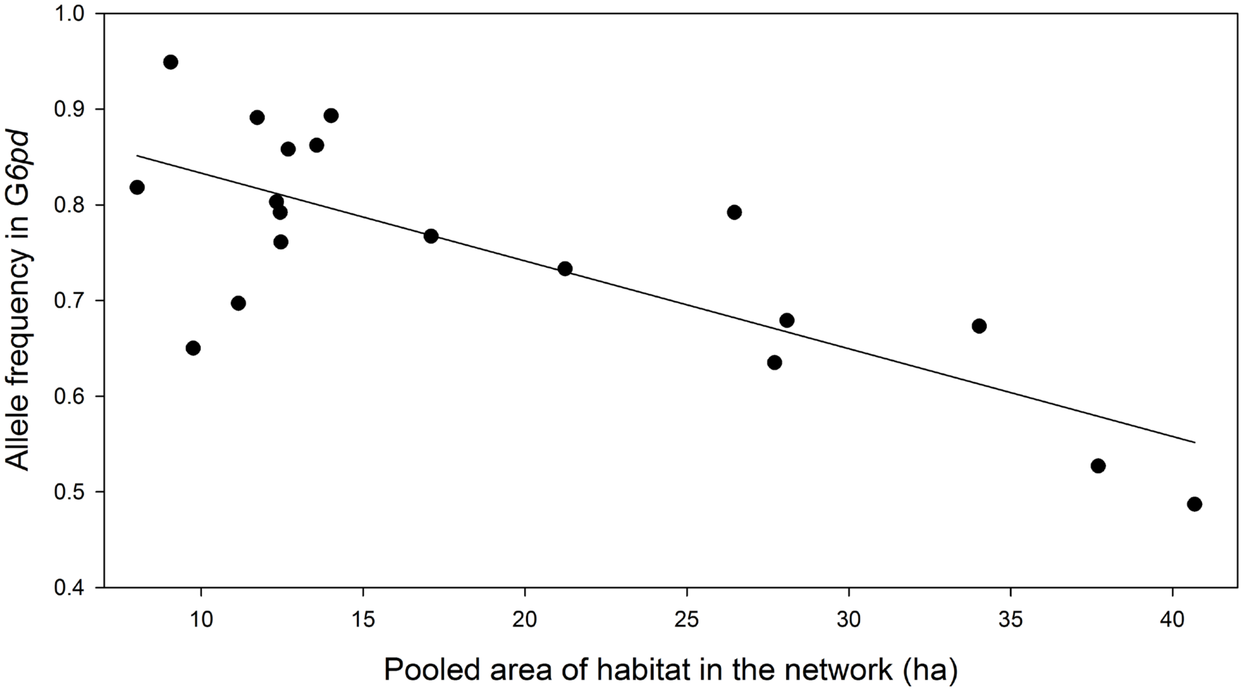

Supplementary Figure 5. Effect of the pooled area of habitat in the network on allele frequency in a SNP Mc1:470:37074 in the gene *G6pd*. The pooled area of habitat explains 57% of variation in allele frequency. Just like in the case of the SNP pgi:c.331A>C, the effect of the pooled area of habitat is due to the number of patches and the average patch area in the network, which both have significant effects on allele frequency and together explain variation in allele frequency as well as the pooled area of habitat (F-test: $F_{1,17} = 24.83$, $P = 0.0001$, $n = 19$).

Supplementary Tables

Supplementary Table 1. Logistic regression model for the probability of larval group detection.

Variable	mean	s.d.	Cr.I.-95%
Intercept	-0.03	0.20	-0.42 – 0.36
log (Area)	-0.36	0.06	-0.49 – -0.25
log (Larval group count)	0.34	0.07	0.21 – 0.41

The mean, standard deviation and 95% credible intervals are reported on the logit-scale. Larval group detection probabilities decrease with patch area and increase with total larval group count (n = 200).

Supplementary Table 2. Logistic regression model for non-detection of occupied patches.

Variable	mean	s.d.	Cr.I.-95%
Intercept	-0.48	0.49	-1.42 – 0.46
log (Area)	0.25	0.18	-0.09 – 0.60
log (Larval group count)	-0.92	0.27	-1.46 – -0.44

The mean, standard deviation and 95% credible intervals are reported on the logit-scale. The probability of not detecting a patch as occupied rapidly decreases with increasing population size, while patch area shows no consistent effect (n = 200).

Supplementary Table 3. Poisson regression model for the effect of non-detection on population size.

Variable	mean	s.d.	Cr.I.-95%
Intercept	2.69	0.03	2.64 – 2.75
log (Area)	0.62	0.02	0.58 – 0.66
Non–detection	-1.22	0.14	-1.52 – -0.95
log (Area) X Non-detection	-0.26	0.09	-0.43 – -0.10

The mean, standard deviation and 95% credible intervals are reported on the log-scale. The population sizes of occupied patches are strongly determined by patch area. The population sizes of patches that were not detected during initial surveys are significantly smaller than those of detected patches of similar size, and the difference in population size between detected and non-detected populations increases with increasing patch size (n = 200).

Supplementary Table 4. Linear regression model for metapopulation size ($\hat{p}_\lambda$) in networks below the extinction threshold (δ = 5.47).

Variable	estimate	s.e.	t_{89}	P
Constant	0.03	0.031	0.92	0.358
Network connectivity	0.01	0.003	3.13	0.002
Metapopulation capacity	0.04	0.012	3.36	0.001

The explanatory variables are network connectivity and metapopulation capacity. The model explains 17% of variation in $\hat{p}_\lambda$ among the networks (F-test: $F_{2,89}$ = 10.56, P = 0.001, n = 92).

Supplementary Table 5. Information on the 19 SNPs analyzed in this study.

SNP ID	Number of networks	Number of samples	Chromosome	Scaffold	Position	Gene ID	Protein description
Mc1:1064:194473	23	6864	2	scaffold1064	194473	MCINX000218	Trypsin-like protease
Mc1:1353:15434	19	6368	15	scaffold1353	15434	MCINX001487	Troponin-T
Mc1:1364:19131	23	6399	18	scaffold1364	19131	MCINX001528	Hemolymph proteinase 5
Mc1:1524:25351	19	6271	31	scaffold1524	25351	MCINX002265	Leucyl aminopeptidase
Mc1:1621:69548	23	6894	11	scaffold1621	69548	MCINX002853	Endocuticle structural glycoprotein SgAbd-8
Mc1:1687:14486	21	6562	1	scaffold1687	14486	MCINX003215	Flightin
Mc1:1785:58421	19	6456	10	scaffold1785	58421	MCINX003735	Heat shock cognate protein 70
Mc1:1791:57299	18	6162	1	scaffold1791	57299	MCINX003812	Alpha-tubulin N-acetyltransferase
Mc1:1884:92472	18	5710	1	scaffold1884	92472	MCINX004366	Triosephosphate isomerase
Mc1:2404:32920	18	6117	1	scaffold2404	32920	MCINX006747	OCIA domain-containing protein 1
Mc1:2760:8235	23	6883		scaffold2760	8235	MCINX008073	Vitellin-degrading protease precursor
Mc1:31:114406	18	6012	1	scaffold31	114406	MCINX009142	Stretchin-Mlck
Mc1:3283:19949 pgi:c.331A>C	27	7068	25	scaffold3283	19949	MCINX009374	Glucose-6-phosphate isomerase
Mc1:337:97709	23	6885	22	scaffold337	97709	MCINX009609	Prophenoloxidase-activating proteinase-3
Mc1:3568:15483	18	6118	1	scaffold3568	15483	MCINX009942	Leucyl aminopeptidase-like protein
Mc1:4593:27912	19	6403		scaffold4593	27912	MCINX011658	Cytochrome P450 337
Mc1:470:37074	19	6244	2	scaffold470	37074	no gene model	Glucose-6-phosphate 1-dehydrogenase
Mc1:6658:103082	23	6832	11	scaffold6658	103082	MCINX014139	Serpin 1
Mc1:7849:5756	23	6835	7	scaffold7849	5756	MCINX015187	Chymotrypsinogen-like protein

The columns give the ID of the SNP, the number of networks above the extinction threshold for which a sufficient sample was available (>10 larval family groups), total number of individuals genotyped, the chromosome, the scaffold ID, position of the SNP in the scaffold, the ID of the gene model, and the description of the protein.
